# Interactive effects of neonatal exposure to monosodium glutamate and aspartame on glucose homeostasis

**DOI:** 10.1186/1743-7075-9-58

**Published:** 2012-06-14

**Authors:** Kate S Collison, Nadine J Makhoul, Marya Z Zaidi, Rana Al-Rabiah, Angela Inglis, Bernard L Andres, Rosario Ubungen, Mohammed Shoukri, Futwan A Al-Mohanna

**Affiliations:** 1 Diabetes Research Unit, Department Cell Biology, King Faisal Specialist Hospital & Research Centre, PO BOX 3354, Riyadh, 11211, Saudi Arabia; 2 Department of Biostatistics, Epidemiology and Scientific Computing, King Faisal Specialist Hospital & Research Centre, Riyadh, Saudi Arabia; 3 College of Medicine, Al-Faisal University, Riyadh, Saudi Arabia

**Keywords:** Aspartame, Monosodium Glutamate, Impaired fasting glucose, Insulin tolerance

## Abstract

**Background:**

Recent evidence suggests that the effects of certain food additives may be synergistic or additive. Aspartame (ASP) and Monosodium Glutamate (MSG) are ubiquitous food additives with a common moiety**:** both contain acidic amino acids which can act as neurotransmitters*,* interacting with NMDA receptors concentrated in areas of the Central Nervous System regulating energy expenditure and conservation. MSG has been shown to promote a neuroendocrine dysfunction when large quantities are administered to mammals during the neonatal period. ASP is a low-calorie dipeptide sweetener found in a wide variety of diet beverages and foods. However, recent reports suggest that ASP may promote weight gain and hyperglycemia in a zebrafish nutritional model.

**Methods:**

We investigated the effects of ASP, MSG or a combination of both on glucose and insulin homeostasis, weight change and adiposity, in C57BL/6 J mice chronically exposed to these food additives commencing *in-utero*, compared to an additive-free diet. Pearson correlation analysis was used to investigate the associations between body characteristics and variables in glucose and insulin homeostasis.

**Results:**

ASP alone (50 mg/Kgbw/day) caused an increase in fasting blood glucose of 1.6-fold, together with reduced insulin sensitivity during an Insulin Tolerance Test (ITT) P < 0.05. Conversely MSG alone decreased blood triglyceride and total cholesterol (T-CHOL) levels. The combination of MSG (120 mg/Kgbw/day) and ASP elevated body weight, and caused a further increase in fasting blood glucose of 2.3-fold compared to Controls (prediabetic levels); together with evidence of insulin resistance during the ITT (P < 0.05). T-CHOL levels were reduced in both ASP-containing diets in both genders. Further analysis showed a strong correlation between body weight at 6 weeks, and body weight and fasting blood glucose levels at 17 weeks, suggesting that early body weight may be a predictor of glucose homeostasis in later life.

**Conclusions:**

Aspartame exposure may promote hyperglycemia and insulin intolerance. MSG may interact with aspartame to further impair glucose homeostasis. This is the first study to ascertain the hyperglycemic effects of chronic exposure to a combination of these commonly consumed food additives; however these observations are limited to a C57BL/6 J mouse model. Caution should be applied in extrapolating these findings to other species.

## Background

Aspartame (L-aspartyl-L-phenylalanine methyl ester: ASP) and Monosodium Glutamate (MSG) are commonly consumed food additives which are incorporated into well over 6000 commonly consumed foods, packaged goods and restaurant fare, where they may be ingested together as part of a meal. The low-calorie dipeptide artificial sweetener ASP is rapidly metabolized upon ingestion into its metabolic components phenylalanine, aspartate and methanol, in the ratio of 50:40:10 w/w/w [1]. Recently, hyperglycemia and weight gain was observed in hypercholesterolemic ASP-fed zebrafish [2]; and chronic exposure to dietary ASP over a period of 3 to 4 months has been shown to increase muscarinic receptor (mAChR) density by up to 80% in many areas of the brain, including the hypothalamus, hippocampus and frontal cortex [3]. mAChRs are acetylcholine receptors highly expressed in the hypothalamus [4], and injections of muscarine into the 3^rd^ cerebral ventricle causes an increase in hepatic venous plasma glucose levels in rats [5]. Previous studies have linked phenylalanine consumption with elevated serum insulin and glucagon levels in healthy subjects [6], and artificial sweetener consumption has been associated with a paradoxical increase in body weight in several [7-9], but not all [10] epidemiological studies.

In rodents, neonatal injections of MSG promotes obesity and growth hormone defects together with hyperinsulinemia and elevated corticosterone levels in adulthood [11-14]. This hypothalamic model of obesity may also be induced in the offspring of pregnant dams orally ingesting MSG [15-17]; and studies with radiolabeled ^3^H-glutamate have shown that glutamate given orally to pregnant mice can subsequently be detected in the maternal and fetal brains and kidneys [18]. The mechanism behind the neuroendocrine disturbance caused by MSG is believed to involve the glutamate-induced degeneration of those areas of the immature neonatal brain which are insufficiently protected by a mature blood–brain barrier, including regions which regulate growth and energy metabolism [11-16]. In 1970, the Joint FAO/WHO Expert Committee on Food Additives set an Acceptable Daily Intake (ADI) limit for MSG of 120 mg/Kg body weight [19]. This recommendation was later revised [20], and the Joint Expert Committee on Food Additives (JEFCA) ruled it was not necessary to set a numerical ADI for MSG, which is also included in the FDA’s Generally Regarded As Safe (GRAS) list [21], together with aspartame [22].

Glutamate is one of the most abundant excitatory neurotransmitter in the brain, and glutamate receptors such as the N-methyl D-aspartate (NMDA) receptor are widely dispersed throughout the central nervous system including the amygdala, hippocampus and hypothalamus, where they regulate many vital metabolic and autonomic functions including energy homeostasis [23], glucose sensing [24], and non-insulin mediated hepatic glucose uptake [25]. Maintaining whole-body glucose homeostasis is of vital importance and requires the integration of hormonal and neuronal signals activated by glucose sensors in various parts of the body including the liver, pancreas and brain. The hypothalamic–pituitary–adrenal (HPA) axis is the predominant system involved in glucose homeostasis, augmenting hepatic glycogenolysis and gluconeogenesis, both essential components of the counter-regulatory response to an acute decrease in blood glucose concentration. Studies have shown that during experimental hypoglycemia, levels of the NMDA receptor ligands glutamate and aspartate rise by up to 10-fold in the central nervous system [26], indicative of a pivotal role of the NMDA receptors in glucose regulation. The partial hyperglycemia induced by neonatal treatment with high-dose injections of MSG is believed to be due to glutamate-mediated destruction of NMDA-receptor rich neurons in the arcuate nucleus, which leads to a higher level of adipose tissue accumulation with resultant insulin resistance and hyperinsulinemia [27,28]. Abnormal glucose homeostasis may result in hyperglycemia leading to insulin resistance; and the prevalence of insulin resistance and type 2 diabetes is increasing world-wide, particularly in the youth, where it is associated with the rise in obesity [29]. Even a mild state of unchecked hyperglycemia may be indicative of prediabetes, a relatively new diagnosis which is defined as having an impaired fasting glucose (IFG) (glucose level ≥ 100 mg/dL but ≤ 125 mg/dL), or impaired glucose tolerance [30].

Recent evidence suggests that the effects of food additives may be synergistic or additive [31]. Given the widespread availability of ASP and MSG in a vast range of processed foods, beverages and restaurant fare, studies on the effects of chronic exposure to these additives would be a timely addition to our knowledge of how recent nutritional changes may influence health outcomes. We therefore examined the effects of chronic exposure to a combination of the food additives ASP and MSG on glucose homeostasis and weight change, compared to either substance on its own, or an additive-free diet. A random-fed insulin tolerance test was used to investigate glucose homeostasis and insulin sensitivity. Additionally Pearson correlation analysis was used to examine the relationship between variables in insulin sensitivity, adiposity, body weight and other body characteristics.

Because exposure to nutritional and environmental challenges during critical periods of early development can markedly affect metabolism in later life [32], and since differentiation of the rodent neuroendocrine system regulating energy homeostasis begins during gestation and continues for a significant period of time after birth [33], our study animals were exposed to these additives *in utero* via the mother’s diet and throughout the first five months of life, in a 2-factor experimental design similar to our previous studies [34,35]. We selected a dosage of ASP which approximates the recognized acceptable daily intake (ADI), which is currently set at 50 mg/Kg body weight in the USA [36]. Monosodium glutamate was administered at 120 mg/Kg BW. To our knowledge this is the first study to examine the effects of neonatal exposure to ASP and MSG on glucose homeostasis in adulthood.

## Methods

### Animals and diets

C57BL/6 J mice were obtained from the Jackson Laboratory and housed/caged in a controlled environment (Pathogen-free conditions of 12 h light/dark cycle, 22 ± 2 °C), and fed a standard chow diet (F648 Laboratory Animal Pellet Diet, Grain Silos and Flour Mills Organization, Saudi Arabia) as previously described [34,35]. See Table 1 for composition of the Standard Chow. Female breeders were maintained on the standard chow diet until six weeks of age whereupon they were placed on one of four different dietary regimens for an adjustment period of three weeks prior to mating at 9 weeks of age as described previously [34,35]. The four dietary intervention groups were (1) *ad lib* Standard Chow with *ad lib* drinking water (Control diet). (2) *Ad lib* Standard Chow, with *ad lib* drinking water containing 0.75 g/L monosodium glutamate (MSG diet: L -Glutamic acid monosodium salt hydrate; catalog G1626 Sigma Aldrich). (3) *Ad lib* Standard Chow, with *ad lib* drinking water containing 0.25 g/L aspartame (ASP diet: Asp-Phe methyl ester, catalog A5139 Sigma Aldrich). (4) *Ad lib* Standard Chow, with *ad lib* drinking water containing 0.25 g/L ASP and 0.75 g/L monosodium glutamate (MSG + ASP diet). After the 3-week period of adjustment to the respective diets, 18 male and 18 female offspring were bred, weaned and maintained on these diets for the duration of the study. These experimental subjects were derived from between 7 and 10 separate litters per diet / gender group (n = 18). Offspring were weaned at 4 weeks of age and housed, 3 to a cage in an identical manner as described above. Food and fluid intake was monitored in all animals at 7 weeks, and again at 15 weeks of age, by weighing the food pellets and water bottles to the nearest 0.1 g. Mean food/fluid consumption of animals housed 3 to a cage was calculated by subtraction. Average body weight was assessed at 6 & 17 weeks of age. Percentage weight change between these two time-points was calculated as follows:

(1) %weightchange=(weightat17weeks−weightat6weeks)/weightat6weeks*100.

**Table 1:** Composition of the Standard Chow diet used throughout the study

**Ingredients (g/100 g dietary weight)**	**Control**	**MSG**	**ASP**	**MSG + ASP**
Protein (%)	22.5	22.5	22.5	22.5
Carbohydrate (%)	64.2	64.2	64.2	64.2
Fat (%) (Ether extract)	5	5	5	5
Fiber (%)	3	3	3	3
Vitamins, Minerals & Ash (%)	5.3	5.3	5.3	5.3
Energy (kcal/g)	3.36	3.36	3.36	3.36
Aspartame (%)	0	0	0.025	0.025
Monosodium Glutamate (%)	0	0.075	0	0.075

Constituents are expressed as percentage of ration except where otherwise indicated.

Mean ASP and MSG consumption were calculated from the amount of ASP-water and MSG-water consumed, and expressed in mg per Kg body weight. At the conclusion of the study (20 weeks of age), overnight-fasted subjects were euthanized with a mixture of xylazine and ketamine, and the blood was collected by cardiac puncture for analysis of serum components. Concomitantly, the visceral fat (epididymal fat pads, together with the gonadal/ovarian adipose tissue associated with the reproductive organs and the omental-mesenteric fat associated with the digestive organs) was carefully excised, rinsed in PBS buffer, blotted dry and weighed to the nearest 0.01 g. The breeding and care of the animals were in accordance with the protocols approved by the Animal Care and Use Committee of the King Faisal Specialist Hospital & Research Centre.

### Measurement of fasting serum glucose, insulin, and lipid profile

Overnight fasting blood glucose was measured from the tail vein of all of the 17-week old experimental subjects (n = 18 per diet and gender group) using the Ascensia Contour glucometer (Bayer HealthCare, IN, USA). Additionally, Serum Triglyceride (TG), T-CHOL, and HDL-C concentrations were also measured in overnight fasted 17-week old mice using the Reflovet Plus instrument (Roche, F. Hoffmann-La Roche Ltd, Basel, Switzerland) as described in our previous studies [34,35]. Fasting insulin was measured using the murine Insulin ELISA kit from Mercodia (Uppsala, Sweden) according to the manufacturer’s instructions.

### Random fed Insulin tolerance test (ITT)

The effect of dietary ASP and MSG on glucose parameters was determined in the same experimental subjects using a random-fed insulin tolerance test (ITT) obtained at 19 weeks of age (n = 18 per diet and gender group). Prior to the commencement of the ITT all animals had full access to food and water. An intraperitoneal injection of insulin (Sigma, IL) at a dose of 0.75 U/kg body weight was administered, and whole blood glucose levels were measured from the tail vein at 0, 15, 30, 45 and 60 min after injection. During the 60-minute test the animals did not have access to food or water. Assessment of insulin sensitivity was made after calculating the Area Under the Curve for glucose (AUC _GLUCOSE_), the rate of glucose utilization (K_ITT_), and the half-life of glucose levels (T_½_). AUCs were calculated using trapezoidal rule. K_ITT_, defined as the percentage decline in glucose per minute, was calculated from the natural log (ln) of glucose concentrations between time t1 and t2, formula K = 100*[(ln-ln)/(t2-t1)]. The serum T_½_ defined as the time in minutes required for the glucose concentration to be halved, was calculated as: T_½_ = (0.693/k)*100 min [37].

### Statistical analysis

All analyses were performed using SAS® version 9.2 (Cary NC, USA) and SPSS version 13 (Chicago IL, USA). Data presented as mean ± SEM for 6 and 17-week body-weight, serum lipid profile and glucose variables separately in male and female mice (n = 18 per diet and per gender) were analyzed using a two way ANOVA for diet and gender. Fasting blood glucose levels was analyzed using a multiple linear regression model to evaluate the significance of the main effects of the diet groups MSG, ASP and MSG + ASP and their interaction. Means of the response variable (fasting blood glucose) were plotted across the levels of the independent variables (diet groups) [38,39]. A three way ANOVA, using the mixed model in SAS was used to test for significant differences between diet, gender, time points and their interaction during the Insulin Tolerance Test. Pearson correlation analysis was performed to evaluate possible associations between output variables in the insulin tolerance test and parameters relating to body weight, fat deposition and lipid profile. Statistical significance was set at P < 0.05.

## Results

### Effect of aspartame and MSG on body characteristics and lipid profile

**Figure 1:**
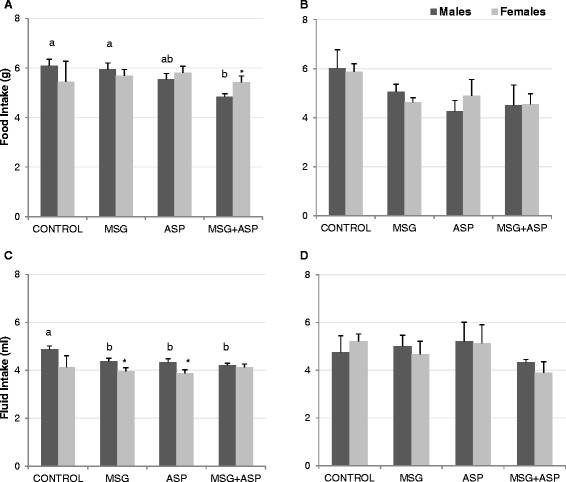
** Food (A,B) and fluid (C,D) intake in mice at 7 (A,C) and 15 weeks of age (B,D), according to diet groups (n = 18).** Statistically significant differences are shown using different letters a,b. Significant gender-wise differences are denoted by * P < .05.

Mean aspartame (ASP) and MSG intake was 55.14 ± 4.74 and 123.44 ± 13 mg/Kg body weight respectively. Food and fluid intakes at 7 and 15 weeks of age are presented in Figure 1. At the earlier time-point, male mice in the MSG + ASP diet consumed slightly less chow than those in the Control and MSG groups, but not statistically significantly less than mice in the ASP diet group (Figure 1A, P < 0.05). Additionally, 7-week old male mice in the Control diet group consumed significantly more fluid than either of the other 3 diet groups (Figure 1C, P < 0.05). We found no differences between food and fluid intake in female 7 week-old mice (Figure 1B & D), and by 15 weeks, no significant differences in food and fluid intake between the four diet groups were apparent in either gender. There were no gender-wise difference between food / fluid intake at 15 weeks of age; however at 7 weeks males in the MSG + ASP diet consumed less chow than females; and males in the MSG and ASP diet groups consumed slightly more fluid than females (P < 0.05).

At 6 and 17 weeks of age, male and female body weight in the MSG + ASP diet group was significantly elevated above the other groups (Table 2, P < 0.01). There was a trend towards higher visceral fat deposition in the MSG + ASP co-treatment group which did not reach statistical significance in both genders; however male mice in all 4 diet groups weighed significantly more than females and the average weight of male visceral fat deposition was twice as high as that of the females in all 4 diet groups (P < 0.01). Fasting serum insulin levels were all within the normal range and there were no significant differences between the four diet groups (Table 2). However, levels of female fasting serum insulin in the control group were significantly lower than males (P < 0.01).

**Table 2:** Effect of aspartame (ASP) and MSG on body weight, fat deposition and lipid profile

**CONTROL**			**MSG**		**ASP**		**MSG + ASP**		**P-value Diet, Gender**
6 Week Body Weight (g)									
male	18.1 ± 0.26	** *a* **	17.41 ± 0.33	** *a* **	17.97 ± 0.22	** *a* **	19.39 ± 0.19	** *b* **	<.0001
female	14.34** ± 0.17		14.15** ± 0.21		14.40** ± 0.15		15.95** ± 0.19		<.0001
17 Week Body Weight (g)									
male	24.92 ± 0.34	** *a* **	25.61 ± 0.32	** *a* **	25.5 ± 0.31	** *a* **	27.84 ± 0.42	** *b* **	<.0001
female	19.24** ± 0.19		18.70** ± 0.19		19.66** ± 0.26		21.61** ± 0.23		<.0001
Weight Change (%)^†^									
male	37.79 ± 1.18^a^		46.46 ± 2.28^b^		42.03 ± 1.38^ab^		43.7 ± 2.04^ab^		0.06
female	34.41 ± 1.47		33.64** ± 1.28		36.67* ± 1.7		35.56** ± 1.12		<.0001
Visceral Fat (g)									
male	0.31 ± 0.02		0.34 ± 0.03		0.34 ± 0.03		0.38 ± 0.03		0.189
female	0.15** ± 0.02		0.14** ± 0.01		0.13** ± 0.01		0.18** ± 0.02		<.0001
Insulin (uIU/mL)									
male	8.76 ± 1.16		8.67 ± 1.7		5.43 ± 1.02		9.67 ± 1.83		0.197
female	4.61** ± 0.74		5.23 ± 0.65		4.34 ± 1.18		6.07 ± 1.20		<0.001
TG (mg/dL)^†^									
male	124.56 ± 6.1^a^		92.88 ± 4.45^b^		95.97 ± 5.61^b^		125.39 ± 2.91^a^		<.0001
female	123.11 ± 5.05^a^		84.47 ± 4.56^b^		93.54 ± 5.59^b^		93.27** ± 5.03^b^		<0.01
T-CHOL (mg/dL)									
male	133.33 ± 2.26	** *a* **	123.94 ± 1.19	** *b* **	118.17 ± 1.2	** *c* **	123.56 ± 1.01	** *b* **	<.0001
female	133 ± 1.81		123.56 ± 1.2		122.33** ± 0.81		124.5 ± 0.92		0.276
HDL-C (mg/dL)									
male	87.48 ± 5.24	** *a* **	71.19 ± 3.21	** *b* **	83.36 ± 2.39	** *ab* **	73.33 ± 2.52	** *b* **	<.01
female	57.62** ± 3.17		54.46** ± 2.19		51.16** ± 2.2		49.38** ± 1.7		<.0001

Data presented are Means ± SEM (n = 18 per diet and per gender). P-values were calculated using 2-way ANOVA. Significant differences between diets are indicated using different letters in bold ** *abc* **. *,** indicates p-value of < .05 and < .01 for the comparison of males to females within each diet.

Data presented are means ± SEM.

P-values for diet and gender are calculated using two way ANOVA.

Significant differences between diets are shown using different letters ** *abc* **.

^†^ Diet*Gender interaction was found to be significant (P < 0.05). Letters abc indicate significant differences between diets separately for each gender.

*,** indicate p-value of < .05, <.01 for the comparison of males to females within each diet.

Both the MSG and the ASP diet groups exhibited decreased levels of total blood cholesterol (T-CHOL) and TG compared to controls (Table 2, P < 0.05), however the MSG + ASP combination did not further reduce the level of either substance. Further effects of the food additives on the lipid profile is suggested by a lowering of levels of HDL-C by both MSG-containing diets (MSG, and MSG + ASP co-treatment) in males but not females (Table 2, P < 0.05).

### Interactive effects of MSG and ASP on glucose homeostasis

**Figure 2:**
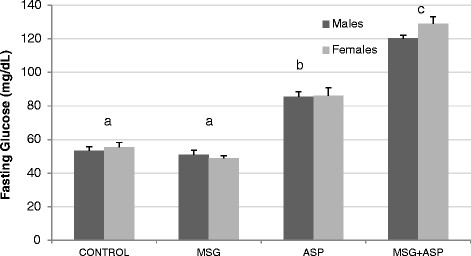
** Effect of aspartame (ASP) and MSG on fasting glucose levels in C57Bl/6 J mice.** Statistically significant differences are shown using different letters abc (n = 18 per diet and per gender).

In addition to the effect of the MSG + ASP combination treatment on body weight and fat deposition, fasting blood glucose levels differed significantly between the 4 diet groups. Treatment with ASP alone raised levels 1.6-fold compared to control; whereas levels in the MSG + ASP group increased by 2.25-fold in males and 2.3-fold in females, suggesting a synergistic effect of MSG on ASP-induced hyperglycemia (Figure 2, P < 0.001). In order to confirm the nature of the interaction, we used a multiple linear regression model which indicated that the diet combination MSG + ASP acted synergistically in elevating fasting blood glucose levels. The average response across the levels of the diet groups were non parallel lines proving the presence of synergy (co-directional interaction). The result of the analysis indicated that ingestion of the combination of food additives elevates fasting blood glucose levels more than the sum of the individual diets ( Additional file 1: Figure 1S, P < 0.01). Interestingly, levels of fasting blood glucose were almost identical in both genders for all four diet groups, indicating that the mechanism behind the ASP-induced glucose elevations is common to both genders.

**Figure 3:**
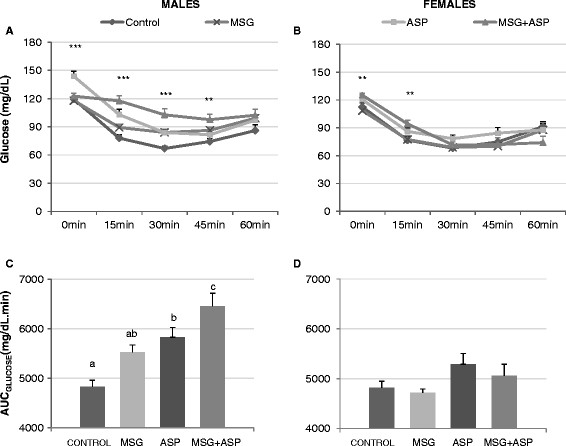
**Random-fed insulin tolerance test. Glucose levels following insulin challenge in (A) male mice and (B) female mice at 0,15,30,45 and 60 minutes after challenge.** Dissimilar means are denoted by **,*** indicating p-values of < .01 and < .001. Area Under the Curve (AUC _GLUCOSE_) as a measure of insulin resistance in (**C**) males and (**D**) females. Statistically significant differences are shown using different letters abc.

In order to examine the effect of the food additive treatment in more detail, we performed a random-fed insulin tolerance test (ITT) on these mice at 19 weeks of age. The ITT demonstrated insulin resistance in both male ASP-containing diet groups compared to control (Figure 3A, P < 0.01). Area Under the Curve analysis of glucose levels (AUC_GLUCOSE_ ) showed that the ASP-containing diets (ASP and MSG + ASP) raised insulin-challenged blood glucose levels significantly higher than the other two diet groups, suggesting impairment of glucose homeostasis in these mice (Figure 3C, P < 0.01). There was a similar trend in females which did not reach statistical significance (Figure 3B & D). In both MSG-containing diet groups (MSG and MSG + ASP), the female AUC _GLUCOSE_ was significantly lower than males, suggesting a higher insulin-stimulated glucose response (P < 0.05). The glucose disappearance coefficient K_ITT;_ a measure of insulin resistance, was significantly decreased by MSG + ASP co-treatment in both genders (Table 3, P < 0.01). Additionally, the half-life of blood glucose (T_½_) was raised by more than double by the MSG + ASP treatment in male subjects, indicative of longer periods of insulin-stimulated hyperglycemia following chronic treatment with both food additives combined. MSG treatment alone had no apparent effect on either AUC _GLUCOSE,_ K_ITT_ or the half-life of glucose during the ITT.

**Table 3:** **AUC**_**GLUCOSE**_**, K**_** ITT **_**and T **_**½**_** values during a random-fed insulin tolerance test administered to male and female C57Bl/6 J mice (n = 18 per gender)**

	**CONTROL**		**MSG**		**ASP**		**MSG + ASP**		**p-value**
	**MEAN**	**SEM**	**MEAN**	**SEM**	**MEAN**	**SEM**	**MEAN**	**SEM**	
Basal Glucose (mg/dL)									
male	119.36^a^	2.80	117.92^a^	4.71	143.72	5.41^b^	122.64^a^	2.90	<.0001
female	112.39^ab^	4.49	108.50^a^	1.96	120.33^ab^**	4.94	125.08^b^	2.30	<.01
AUC_GLUCOSE_ (mg/dL.min)									
male	4829.58^a^	131.96	5522.29^ab^	152.69	5829.79^bc^	197.07	6454.79^c^	266.98	<.0001
female	4819.93	190.36	4717.92**	76.45	5288.33	217.38	5057.92**	233.79	0.156
K. _ITT_ (%/min)									
male	2.92^a^	0.28	2.10^ab^	0.20	2.32^a^	0.29	1.15^b^	0.26	0.001
female	2.60	0.37	2.28	0.26	2.40	0.19	1.95*	0.26	0.436
T½_GLUCOSE_ (min)									
male	39.69^a^	14.05	37.91^a^	3.84	51.61^ab^	16.63	105.56^b^	23.72	0.022
female	39.02	6.38	41.54	7.66	32.36	2.90	92.55	36.44	0.105

Significant differences between diets are indicated by different letters abc. *, ** indicate differences between males and females within each diet P < 0.05 and 0.01 respectively.

P-values for comparison of diets are calculated using one way ANOVA, dissimilar means are denoted by different letters abc.

*,** indicate p-value of < .05, <.01 when comparing males to females within each diet group.

In the control diet group, males and females shared almost identical AUC _GLUCOSE,_ K_ITT_ and T½ values, suggesting that glucose homeostasis was similar in both genders under normal conditions (Table 3). Additionally even though the glucose half-life increased in response to ASP treatment, and the K_ITT_ decreased, the levels of these parameters were almost identical in both genders, indicating a shared mechanism for the ASP-induced impairment in glucose homeostasis (Table 3).

Taken together our results suggest that ASP treatment increased fasting blood glucose levels, and the combination of ASP plus MSG caused further impairment of glucose homeostasis in both genders, which was accompanied by an increased body weight apparent at both 6 and 17 weeks of age. It also suggests that MSG appears to interact with ASP in raising blood glucose levels to prediabetes levels (≥ 100 mg/dL) in these mice.

### Correlation analysis

In order to further understand the underlying mechanisms behind the diet-induced weight gain and glucose deregulation, and the gender-specific differences in the observed outcome parameters, we performed Pearson correlation analysis between ASP and MSG consumption, variables in the ITT (AUC _GLUCOSE_, K _ITT_, T ½), and the body characteristics listed in Table 2. Irrespective of diet, in both genders we identified a strong correlation between ASP intake and fasting blood glucose levels in males and females (Table 4, P < 0.01). MSG intake correlated with fasting blood glucose to a lesser extent but was still highly significant in males and females. Further positive correlations between ASP intake were found with body weight at both 6 and 17 weeks of age, and with random-fed glucose levels. Area under the curve for glucose (AUC _GLUCOSE_) during the ITT, together with the half-life of glucose, correlated positively with ASP intake. Conversely, MSG intake was associated with weight at 17 weeks of age, % weight change in males; and both 6-week and 17-week body weight in females. Interestingly ASP intake correlated negatively with total cholesterol levels in both genders, suggesting that higher levels of ASP intake are associated with lower levels of blood lipids. We also found a correlation between body weight at 6 weeks, and body weight and fasting blood glucose levels at 17 weeks, suggesting that early body weight may be a good predictor of glucose homeostasis in later life. Additionally, visceral fat accumulation correlated with random blood glucose in males and fasting glucose levels in female mice. Furthermore in both genders, percentage weight change correlated positively with visceral fat deposition together with fasting and random blood glucose levels, suggesting that increased weight is associated with increased adiposity and glucose deregulation in these mice. Moreover in both genders, early body weight correlated negatively with percentage weight gain, indicating that a lower post-weaning body weight may be indicative of larger weight gains in adulthood, and vice versa.

**Table 4:** Pearson correlation analysis of insulin resistance parameters, ASP and MSG intake and body characteristics in C57BL/6 J mice

** *Males* **	**ASP Intake (mg/kg)**	**MSG Intake (mg/kg)**	**6 Week Body Weight (g)**	**17 Week Body Weight (g)**	**Weight Change (%)**	**Visceral Fat (g)**	**Fasting Glucose (mg/dL)**	**T-CHOL (mg/dL)**	**HDL-C (mg/dL)**	**TG (mg/dL)**	**Random Glucose (mg/dL)**	**AUC**_**GLUCOSE**_**(mg/dL.min)**	**K.**_**ITT**_**(%/min)**	**T½**_**GLUCOSE**_**(min)**
**Females**														
ASP Intake (mg/kg)		*−0.150*	0.377**	0.391**	*0.055*	*0.153*	0.723**	−0.473**	*−0.029*	*0.042*	0.360**	0.472**	−0.270*	0.262*
MSG Intake (mg/kg)	0.181		*0.214*	0.394**	0.289*	*0.156*	0.297**	*−0.066*	−0.346**	*0.060*	*−0.195*	0.377**	−0.384**	*0.176*
6 Week Body Weight (g)	0.510**	0.467**		0.698**	−0.371**	*0.076*	0.442**	*−0.221*	*0.028*	0.379**	*−0.049*	−0.257*	−0.231*	*−0.022*
17 Week Body Weight (g)	0.607**	0.414**	0.812**		*0.219*	0.261*	0.606**	−0.286*	*−0.042*	0.228*	*0.092*	*−0.087*	−0.413**	*−0.119*
Weight Change (%)	0.151	−0.090	−0.299*	0.435**		0.450**	0.282*	*0.064*	*0.070*	*0.082*	0.294*	*0.124*	*−0.210*	*−0.078*
Visceral Fat (g)	0.078	0.222	0.340**	0.400**	0.265*		*0.163*	*−0.012*	0.236*	*0.035*	0.335**	*0.150*	*−0.104*	*0.032*
Fasting Glucose (mg/dL)	0.809**	0.378**	0.587**	0.790**	0.378**	0.269*		−0.343**	*−0.115*	*0.060*	0.321**	*−0.195*	−0.417**	*−0.153*
T-CHOL (mg/dL)	−0.423**	−0.222	0.030	0.031	0.142	0.196	−0.058		*0.027*	*0.174*	*−0.123*	*−0.056*	*0.136*	*0.074*
HDL-C (mg/dL)	−0.263*	−0.172	−0.098	−0.216	−0.237	0.014	−0.153	0.050		0.312**	*0.161*	*−0.189*	0.315**	*0.200*
TG (mg/dL)	−0.245*	−0.330**	−0.053	0.015	0.161	−0.241	−0.100	0.279*	0.075		−0.369**	*−0.214*	*−0.102*	*0.117*
Random Glucose (mg/dL)	0.241	−0.102	0.267*	0.459**	0.306*	0.175	0.446**	0.318*	−0.126	0.084		*0.036*	*0.194*	** *−0.003* **
AUC_GLUCOSE_ (mg/dL.min)	0.235	−0.126	−0.071	0.005	0.073	0.150	0.080	0.275*	0.173	0.076	0.338**		*−0.108*	−0.270*
K._ITT_ (%/min)	−0.214	−0.202	−0.017	−0.068	−0.085	−0.184	−0.145	0.139	−0.090	0.107	0.329*	−0.080		*0.194*
T½_GLUCOSE_ (min)	0.135	0.266*	0.214	0.218	0.041	0.235	0.255*	0.077	−0.032	−0.101	0.038	−0.066	−0.400**	

Significant correlations are indicated in bold by ** at the 0.01 level and * at the 0.05 level (2-tailed). Correlations in males (n = 72) are indicated in *italics* (top right), and correlations in female subjects (n = 72) are shown in the bottom left of the table.

Significant correlations are indicated in **bold** by ** at the 0.01 level and * at the 0.05 level (2-tailed).

Correlations in males are indicated in *italics*.

In males but not females, 6-week body weight negatively correlated with AUC _GLUCOSE_ and the glucose disappearance coefficient K _ITT_ (Table 4). In females however, the negative correlations were weaker and without statistical significance. Additionally, further analysis of male mice indicated that weight change correlated positively with visceral fat and fasting glucose levels, and negatively with body weight at 6 weeks of age. Triglyceride (TG) levels correlated with early and adulthood body weights. A subtle difference between the genders was that visceral fat deposition correlated with early and adult body weight together with percentage weight gain in females, whereas male adiposity only correlated with adulthood body weight and percentage weight gain.

Correlation analysis was further extended to examine the associations between these covariates when stratified by diet and gender. Additional file 2: Table S1 shows a diet-stratified correlation matrix. In both genders, the rate of glucose utilization (K_ITT)_ negatively correlated with the half-life of glucose (T_½_) in all four diet groups ( Additional file 2: Table S1A-D, P < 0.05). In all the diet groups, 6-week body weight correlated significantly with 17-week body weight except for males in the MSG + ASP group, ( Additional file 2: Table S1A-D). There were several positive correlations between visceral fat and body weight in both the MSG and MSG + ASP diet groups (both genders: Additional file 2: Table S1B and 1D P < 0.05). In males, fasting blood glucose correlated with MSG intake in the MSG diet group ( Additional file 2: Table S1B), and with both MSG and ASP intake in MSG + ASP diet group ( Additional file 2: Table S1D). Visceral fat deposition correlated with MSG intake in both genders in the MSG diet group ( Additional file 2: Table S1C), but only in females consuming both additives ( Additional file 2: Table S1D).

In summary, our data suggests that exposure to a combination of dietary ASP and MSG may promote an increase in body weight, adiposity and markers of developing insulin resistance, including fasting blood glucose elevations and impairment in insulin sensitivity. Early body weight correlated with impairment of glucose homeostasis, and was a predictor of weight gain and adiposity in adulthood.

## Discussion

The present study suggests that neonatal exposure to aspartame (ASP), or a combination of MSG and ASP, together with continued exposure to these additives for the first five months of life can markedly influence glucose homeostasis in young adult male and female C57BL/6 J mice. Our data confirms previous findings that ASP treatment promotes hyperglycemia and weight gain in hypercholesterolemic zebrafish [2]. The timing of exposure to these food additives appears to be critical in determining the outcome, since it has previously been shown that acute administration of a high dose of ASP to adult diabetic rats had no effect on plasma glucose levels [40]. In our study, exposure commenced *in utero* via transfer of amino acids through the placenta, and continued during breast-feeding and through to adulthood via the drinking water consumed daily. Experimentally, this design resembles the patterns of exposure to food additives which may occur in other species, such as primates.

Several studies have shown that nutrition in neonatal and fetal life may lead to related disorders in adulthood such as cardiovascular disease and obesity [33]. Furthermore, studies have suggested that in rodents, chronic treatment with ASP [3,41] or MSG [42], or prenatal exposure to these additives [43,44] may cause behavioral differences and learning impairment, suggesting the possibility of an effect on centers of learning and development in the brain [1], which are intricately linked to insulin and glucose homeostasis [45]. Glutamate derived from dietary MSG could cause a rapid spiking of plasma glutamate levels compared to similar amounts of glutamate bound to other amino acids in dietary proteins [46]; and since ASP is also metabolized rapidly into its two amino acids phenylalanine and aspartate, which are normally only found in the bound form in dietary protein, concerns emerged over potential neurotoxicity arising from the interaction between ASP and MSG [47,48].

The immediate metabolic products of ASP are phenylalanine, aspartate and methanol, in the ratio of 50:40:10w/w/w [1]. Phenylalanine metabolism in the body can follow one of two pathways: conversion into tyrosine by the hepatic enzyme phenylalanine hydroxylase (PAH); alternatively it can compete with other large neutral amino acids for binding sites on the large neutral amino acid transporter (NAAT), to be carried across the blood–brain barrier [1]. Both phenylalanine and tyrosine are intimately involved in the production of several key neurotransmitters such as dopamine, norepinephrine and serotonin. Furthermore, phenylalanine also plays a role in amino acid metabolism and protein structuring in all body tissues. It has previously been shown that a single dose of 200 mg/Kg ASP by gavage increased rodent plasma phenylalanine and tyrosine levels by 62% and 142% respectively [49]. Elevated levels of ASP-derived phenylalanine could potentially accumulate in the brain owing to its ability to compete with tyrosine for the NAAT at the blood–brain barrier. This in turn could lead to changes in the regional brain concentrations of these neurotransmitters; and indeed, a dose-dependent reduction in levels of dopamine, serotonin and norepinephrine, together with increased levels of oxidative stress markers has recently been demonstrated in the brains of ASP-treated mice [50]. Thus, ASP and its metabolites have the potential to disrupt a wide range of cellular processes including neuroendocrine balances. Further elucidation of these mechanisms is discussed in greater detail in a comprehensive review by Humphries *et al*[1].

Previous research has shown that hyperphenylalaninemic rodents have lower brain weights [51] together with impaired myelinogenesis [52]. Interestingly, increased hepatic glucose production and plasma glucose levels have been reported in rats challenged with an acute load of phenylalanine [53]. Further evidence for a mechanism for the effects of ASP on glucose homeostasis is provided by a study which showed that ASP may increase muscarinic receptor density by up to 80% in many areas of the brain, including the hypothalamus [3]. Moreover, microdialysis studies have shown that activation of muscarinic and ACh-receptive neurons (mAChRs) in the hypothalamus caused an elevation in rodent plasma glucose levels, which could be reduced by the mAChRs antagonist atropine [54], suggesting a role for hypothalamic mAChRs in glucose homeostasis.

In our study, ASP (55.14 mg/Kg BW/day) raised fasting blood glucose levels by 1.6-fold, whereas a combination of ASP and MSG (123.44 mg/Kg BW/day) further raised fasting glucose to prediabetic levels in both genders. The similarity in response to ASP and MSG in terms of glucose homeostasis is particularly striking, and points to a shared mechanism between the genders. It is tempting to speculate that the interaction between ASP and MSG may converge at the level of the NMDA receptor for which glutamate and aspartate are both ligands; however the situation is likely to be far more intricate. Glutamate is the most abundant excitatory neurotransmitter and plays a pivotal role in the formation of synapses, integration of convergent signals, the establishment of N-methyl-D-aspartate (NMDA) receptor-dependent long term potentiation, and several critical autonomic functions including appetite regulation and thermogenesis [55]. However, elevated levels of glutamate have been shown to cause selective neuronal damage in the brains of infant mice, in particular the highly vascularized areas located outside the blood–brain barrier such as the median eminence and the hypothalamic arcuate nucleus [56,57]. Earlier research has established that the minimum concentration of MSG required to cause injury to murine hypothalamic neurons is 200 mg/Kg BW [58]. At this concentration, plasma glutamate levels spiked by 16-fold after 15 minutes of exposure; and resulted in increased NMDA receptor expression together with accumulation of glutamate in the hypothalamic tanycytes [58]. However, even nonexcitotoxically-derived glutamate may affect neurotransmission and correlates of brain glutamatergic function [59]; this notion is supported by the finding that elevated levels of glutamate have been shown to modulate the expression of glutamate transporters (GLT-1 and GLAST) without promoting overt cellular injury [59-62].

Existing evidence using 3 H-glutamate radiolabeled tracer studies suggest that in rodents, glutamate can cross the placental barrier and accumulate in the immature fetal central nervous system [44]. However whereas it is generally agreed that aspartate crosses the placenta only to a limited degree [63], phenylalanine is actively transported across the placenta [64]; resulting in an increase in phenylalanine at the expense of the maternal concentration [65]. In rodents, phenylalanine is also readily converted into the neurotransmitter precursor tyrosine by the hepatic enzyme phenylalanine hydroxylase (PAH) [66]; but if the activity of this enzyme is reduced or absent, the high levels of accumulated phenylalanine may be converted into other metabolites such as β-Phenylpyruvate [67,68], which are known to interfere with normal glucose metabolism [68,69]. Crucially, studies have shown that in rodents, PAH activity is absent until a late stage of gestation, or shortly after birth [70-72]; and experimentally induced hyperphenylalaninemia in rats diminishes cerebral glycolysis by inhibiting hexokinase and pyruvate kinase [73], leading to impairment of glucose metabolism in the hyperphenylalaninemic rodent brain [74,75]. Taken together, this evidence suggests a number of potential mechanisms which could be responsible for the perturbation in glucose homeostasis that we observed. Further studies are required to explore these possibilities in more detail.

One further observation from our study shows a reduction in total cholesterol (T-CHOL) levels in both male and female C57BL/6 J mice following ASP and MSG exposure, together with a lowering of triglyceride levels in female mice in the 3 diet groups compared to Control. A slight reduction in serum cholesterol has also previously been noted in Wistar rats exposed to ASP at a concentration of 4 g/Kg/day (approximately 80 times the ADI for ASP) for a period of approximately 24 months [76]. In humans, ingestion of 150 mg/Kg bw for six weeks also lowered serum cholesterol and phospholipids [77], although the relevance of this observation to the present rodent study is questionable. Additionally, the mechanism behind this hypolipidemic effect remains to be established.

Our correlation analysis revealed a positive association between ASP intake and body weight in both genders. As mentioned earlier, artificial sweetener intake has been associated with increased weigh gain in several, but not all human epidemiological studies in the past [7-10]. Our correlation analysis also indicated that early body weight may be a good predictor of glucose homeostasis in later life. We found positive correlations between blood glucose levels and body weight, and between glucose levels and visceral fat deposition. Previous studies have indicated that glucose homeostasis in adulthood is programmed during gestation [78-80] at a time when the hypothalamus is vulnerable to excitotoxic injury [1-6]; and several prospective studies have found associations between early life adiposity and weight gain with the development of diabetes in later life [81]. Our study concluded when the animals reached 5 months of age (mature adulthood). It would be of interest to ascertain whether the ASP-induced impairment of glucose homeostasis would still be apparent at a later time-point, since it is known that glucose homeostasis deteriorates with aging in C57Bl/6 J mice.

## Conclusions

Our experimental model provides a valid means of investigating the interaction between two ingested food additives, and demonstrates how ASP and MSG may interact *in vivo* to promote weight gain and impair glucose homeostasis. Studies of this nature are of relevance to the human population, where consumption of food additives are almost impossible to avoid in today’s diet. However care must be taken in extrapolating our findings further, and it will be important to investigate the interactions between MSG and ASP in other species.

## Abbreviations

ASP = aspartame; ADI = Acceptable Daily Intake; ITT = Insulin tolerance test; AUC GLUCOSE = Area Under the Curve for glucose; T½ = Half-life of glucose; K ITT = Glucose disappearance rate; MSG = Monosodium Glutamate; TG = Triglyceride; T-CHOL = Total cholesterol; HDL = High density lipoprotein cholesterol.

## Competing interests

The authors declare that they have no competing interests.

## Authors’ contributions

KSC conceived the study, designed the experiments and drafted the manuscript. NJM, RA-R, AI, BA, and RU performed the experiments. MZZ, MS,NJM and KSC analyzed the data. FAA & MZ helped draft the manuscript. We are grateful for continuous discussions and support from FAA. All Authors read and approved the final manuscript.

## Supplementary Material

Additional file 1 Figure S1. Main effect interaction of the diet group MSG, ASP and MSG + ASP (mean ± SEM, n = 36 per diet group). Click here for file

Additional file 2 Table S1. Correlation analysis of insulin resistance parameters, ASP and MSG intake and body characteristics in Control (A), MSG (B), ASP (C) and the MSG + ASP diet (D) mice. Significant correlations are indicated in bold by ** at the 0.01 level and * at the 0.05 level (2-tailed). Correlations in males (n = 18 per diet group) are indicated in *italics* (top right), and correlations in female subjects are shown in the bottom left of the table. Click here for file
