# Supplementary figures and images for: Interactive effects of neonatal exposure to monosodium glutamate and aspartame on glucose homeostasis
Source: Nutr Metab (Lond). 2012 Jun 14;9:58. doi: 10.1186/1743-7075-9-58 (PMC3466134; doi:10.1186/1743-7075-9-58)

Supplemental Figure 1.

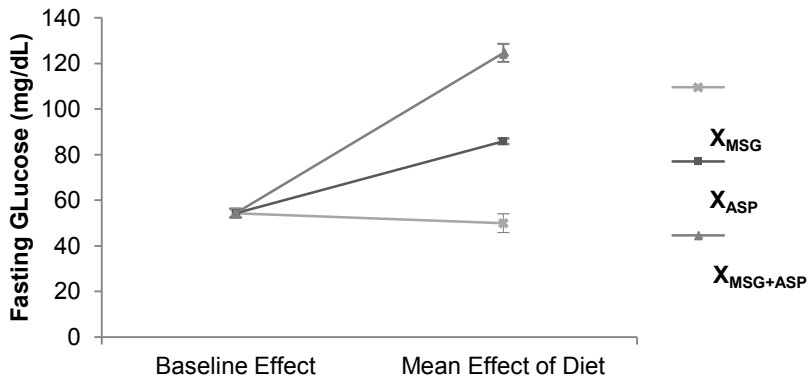

Supplement: Additional file 1 — Figure S1. Main effect interaction of the diet group MSG, ASP and MSG + ASP (mean ± SEM, n = 36 per diet group). [file 1743-7075-9-58-S1.pdf]
