# Supplementary material for: Interactive effects of neonatal exposure to monosodium glutamate and aspartame on glucose homeostasis
Source: Nutr Metab (Lond). 2012 Jun 14;9:58. doi: 10.1186/1743-7075-9-58 (PMC3466134; doi:10.1186/1743-7075-9-58)
Supplement: Additional file 2 — Table S1. Correlation analysis of insulin resistance parameters, ASP and MSG intake and body characteristics in Control (A), MSG (B), ASP (C) and the MSG + ASP diet (D) mice. Significant correlations are indicated in bold by ** at the 0.01 level and * at the 0.05 level (2-tailed). Correlations in males (n = 18 per diet group) are indicated in italics (top right), and correlations in female subjects are shown in the bottom left of the table. [file 1743-7075-9-58-S2.pdf]

Supplemetal Table 1 A. Correlation analysis of insulin resistance parameters and body characteristics in mice in CONTROL diet group.

Males<br>Females	6 Week Body Weight (g)	17 Week Body Weight (g)	Weight Change (%)	Visceral Fat (g)	Fasting Glucose (mg/dL)	T-CHOL (mg/dL)	HDL-C (mg/dL)	TG (mg/dL)	Random Glucose (mg/dL)	AUC$_{GLUCOSE}$(mg/dL.min)	K.$_{ITT}$ (%/min)	T½$_{GLUCOSE}$ (min)
6 Week Body Weight (g)		***0.809***	*-0.389*	*0.325*	*-0.420*	*-0.431*	*0.369*	*0.269*	*0.130*	*-0.055*	*0.310*	*-0.088*
17 Week Body Weight (g)	**0.518***		*0.227*	*0.184*	*-0.356*	*-0.351*	*0.428*	***0.506****	*0.175*	*-0.054*	*0.336*	*-0.075*
Weight Change (%)	**-0.614****	0.357		*-0.260*	*0.129*	*0.166*	*0.061*	*0.347*	*0.068*	*0.021*	*0.016*	*0.022*
Visceral Fat (g)	0.351	0.412	0.011		*0.267*	***-0.521****	*0.166*	*0.053*	*0.362*	*0.263*	*-0.023*	*-0.135*
Fasting Glucose (mg/dL)	**-0.528***	-0.277	0.320	-0.096		*0.247*	*0.092*	*-0.065*	*0.433*	*0.117*	*-0.005*	*0.054*
T-CHOL (mg/dL)	-0.250	-0.178	0.111	0.053	0.302		*-0.412*	*-0.174*	*0.007*	*-0.288*	*-0.128*	*-0.027*
HDL-C (mg/dL)	0.045	0.148	0.088	-0.024	0.400	-0.001		***0.519****	*0.436*	*0.140*	*0.417*	*0.009*
TG (mg/dL)	-0.302	-0.274	0.076	-0.190	**0.574***	0.039	0.270		*0.033*	*-0.045*	*0.353*	*0.069*
Random Glucose (mg/dL)	-0.492	-0.551	0.152	0.641	0.532	0.499	0.721	0.426		*0.335*	*0.231*	*-0.260*
AUC$_{GLUCOSE}$(mg/dL.min)	-0.255	-0.197	0.116	**0.999***	0.757	0.627	0.783	0.544	0.427		*-0.381*	***0.469****
K$_{ITT}$ (%/min)	-0.430	-0.278	0.296	-0.725	-0.240	-0.006	-0.159	-0.396	**0.531***	-0.399		***-0.688*****
T½ $_{GLUCOSE}$ (min)	-0.010	-0.134	-0.105	0.970	0.528	0.352	0.495	0.603	-0.393	**0.554***	**-0.761****	

Significant correlations are indicated in **bold** by ** at the 0.01 level and * at the 0.05 level (2-tailed).
Correlations in males are indicated in *italics* .

Supplemetal Table 1B. Correlation analysis of insulin resistance parameters, MSG intake and body characteristics in mice in MSG diet group.

Males / Females	MSG Intake (mg/kg)	6 Week Body Weight (g)	17 Week Body Weight (g)	Weight Change (%)	Visceral Fat (g)	Fasting Glucose (mg/dL)	T-CHOL (mg/dL)	HDL-C (mg/dL)	TG (mg/dL)	Random Glucose (mg/dL)	$AUC_{GLUCOSE}$(mg/dL.min)	K_{ITT} (%/min)	$T½_{GLUCOSE}$ (min)
MSG Intake (mg/kg)		0.834**	0.627*	-0.725**	0.522*	0.616*	-0.338	-0.029	-0.065	0.285	0.008	0.076	0.039
6 Week Body Weight (g)	0.770*		0.627*	-0.725**	0.522*	0.615*	-0.338	-0.029	-0.065	0.285	0.007	0.099	-0.132
17 Week Body Weight (g)	0.762**	0.761**		0.078	0.563*	0.586*	-0.343	-0.146	-0.275	0.149	-0.193	0.056	-0.187
Weight Change (%)	-0.698**	-0.698**	-0.070		-0.204	-0.285	0.337	0.019	-0.044	-0.017	-0.084	-0.033	-0.010
Visceral Fat (g)	0.510*	0.510*	0.465	-0.273		0.781**	-0.206	0.072	-0.140	0.350	-0.005	0.180	-0.173
Fasting Glucose (mg/dL)	0.328	0.328	0.284	-0.303	0.542*		-0.191	-0.042	-0.269	0.259	0.013	0.006	-0.193
T-CHOL (mg/dL)	0.028	0.027	0.326	0.103	0.555*	0.089		0.399	0.140	0.264	0.216	-0.190	0.250
HDL-C (mg/dL)	-0.133	-0.133	-0.190	0.141	-0.019	-0.166	-0.203		0.261	-0.089	-0.336	-0.070	0.178
TG (mg/dL)	-0.224	-0.223	0.183	0.375	-0.368	-0.227	0.002	0.252		-0.354	0.149	-0.535*	0.583*
Random Glucose (mg/dL)	0.318	0.318	0.228	-0.274	-0.043	0.043	-0.190	-0.393	-0.242		0.514*	0.797**	-0.707**
$AUC_{GLUCOSE}$(mg/dL.min)	0.329	0.329	0.335	-0.057	0.082	0.550*	-0.155	-0.113	-0.088	0.420		0.274	-0.233
K_{ITT} (%/min)	0.435	0.435	0.099	-0.387	-0.086	0.059	-0.373	-0.582*	-0.397	0.653**	0.310		-0.904**
$T½_{GLUCOSE}$ (min)	-0.342	-0.342	-0.229	0.090	0.199	0.272	0.220	0.315	0.242	-0.457*	-0.124	-0.742**	

Significant correlations are indicated in **bold** by ** at the 0.01 level and * at the 0.05 level (2-tailed).
Correlations in males are indicated in *italics* .

Supplemetal Table 1C. Correlation analysis of insulin resistance parameters and body characteristics in mice in ASP diet group.

Males<br>Females	ASP Intake (mg/kg)	6 Week Body Weight (g)	17 Week Body Weight (g)	Weight Change (%)	Visceral Fat (g)	Fasting Glucose (mg/dL)	T-CHOL (mg/dL)	HDL-C (mg/dL)	TG (mg/dL)	Random Glucose (mg/dL)	$AUC_{GLUCOSE}$(mg/dL. min)	$K._{ITT}$ (%/min)	$T½_{GLUCOSE}$ (min)
ASP Intake (mg/kg)		___0.486*___	___0.682**___	_-0.422_	_0.043_	_-0.217_	_-0.204_	_-0.058_	___0.628**___	_-0.319_	_-0.380_	_-0.175_	_0.356_
6 Week Body Weight (g)	-0.243		___0.680**___	_-0.424_	_0.034_	_-0.219_	_-0.211_	_-0.049_	___0.637**___	_-0.329_	_-0.391_	_-0.165_	_0.369_
17 Week Body Weight (g)	__0.464*__	__0.461*__		_0.376_	_0.101_	_0.351_	_-0.075_	_0.177_	_0.165_	_0.247_	_0.030_	_-0.082_	_0.074_
Weight Change (%)	-0.371	-0.374	__0.650**__		_0.075_	___0.717**___	_0.162_	_0.293_	___-0.599**___	___0.715**___	___0.528*___	_0.111_	_-0.353_
Visceral Fat (g)	0.103	0.106	-0.119	-0.185		_-0.083_	_-0.022_	___0.519*___	_0.164_	_0.083_	_0.104_	_0.139_	_-0.041_
Fasting Glucose (mg/dL)	0.353	0.352	__0.631**__	0.352	-0.068		_-0.320_	_-0.041_	___-0.525*___	___0.598**___	_0.336_	_0.196_	_-0.247_
T-CHOL (mg/dL)	0.043	0.040	0.105	0.073	-0.112	0.188		_0.097_	_-0.380_	_0.439_	___0.487*___	_-0.128_	_-0.277_
HDL-C (mg/dL)	0.417	0.419	0.103	-0.253	0.392	0.207	-0.101		_0.047_	_0.048_	_0.208_	_-0.061_	_0.255_
TG (mg/dL)	-0.330	-0.327	-0.342	-0.086	0.242	-0.420	-0.276	-0.084		___-0.701**___	___-0.606**___	_-0.081_	_0.448_
Random Glucose (mg/dL)	-0.031	-0.033	__0.674**__	__0.736**__	-0.226	__0.738**__	0.310	-0.177	-0.251		___0.793**___	_-0.028_	_-0.349_
$AUC_{GLUCOSE}$(mg/dL.min)	-0.088	-0.090	0.258	0.343	-0.001	-0.081	0.427	-0.218	-0.234	0.297		___-0.464*___	_0.042_
K_{ITT} (%/min)	0.132	0.133	0.067	-0.033	0.406	__0.477*__	0.236	0.296	-0.284	0.383	-0.075		___-0.623**___
$T½_{GLUCOSE}$ (min)	-0.037	-0.038	-0.025	-0.006	-0.354	-0.415	-0.333	-0.216	0.180	-0.434	0.012	__-0.960**__	

Significant correlations are indicated in **bold** by ** at the 0.01 level and * at the 0.05 level (2-tailed).
Correlations in males are indicated in _italics_ .

Supplemetal Table 1D. Correlation analysis of insulin resistance parameters and body characteristics in mice in MSG+ASP diet group.

Males / Females	ASP Intake (mg/kg)	MSG Intake (mg/kg)	6 Week Body Weight (g)	17 Week Body Weight (g)	Weight Change (%)	Visceral Fat (g)	Fasting Glucose (mg/dL)	T-CHOL (mg/dL)	HDL-C (mg/dL)	TG (mg/dL)	Random Glucose (mg/dL)	$AUC_{GLUCOSE}$(mg/dL.min)	K_{ITT} (%/min)	$T½_{GLUCOSE}$ (min)
ASP Intake (mg/kg)		***0.999*****	*0.416*	*0.316*	*-0.309*	*-0.095*	***0.531****	*-0.235*	*-0.211*	*-0.280*	*-0.187*	*-0.057*	*0.206*	*-0.403*
MSG Intake (mg/kg)	**0.999****		*0.416*	*0.368*	*-0.308*	*-0.093*	***0.532****	*-0.236*	*-0.211*	*-0.280*	*-0.185*	*-0.056*	*0.210*	*-0.406*
6 Week Body Weight (g)	**0.788****	**0.788****		*0.368*	*-0.308*	*-0.093*	***0.532****	*-0.237*	*-0.211*	*-0.281*	*-0.185*	*-0.056*	*0.209*	*-0.406*
17 Week Body Weight (g)	**0.752****	**0.752****	**0.752****		***0.771*****	***0.511****	***0.644*****	*-0.289*	*0.022*	*0.027*	*0.201*	*0.415*	*-0.042*	*0.040*
Weight Change (%)	**-0.488***	**-0.488***	**-0.488***	0.208		***0.581****	*0.291*	*-0.132*	*0.179*	*0.223*	*0.336*	***0.478****	*-0.195*	*0.320*
Visceral Fat (g)	**0.671****	**0.671****	**0.671****	**0.523***	-0.393		*0.040*	*0.231*	***0.523****	***0.535****	*-0.068*	*0.060*	*0.030*	*-0.096*
Fasting Glucose (mg/dL)	-0.298	-0.296	-0.297	0.095	**0.556***	-0.222		***-0.700*****	*-0.212*	*-0.072*	*0.171*	*0.456*	*0.469*	*-0.428*
T-CHOL (mg/dL)	0.324	0.324	0.323	0.083	-0.387	0.270	-0.221		*0.292*	*0.280*	*0.090*	*-0.338*	*-0.312*	*0.243*
HDL-C (mg/dL)	-0.159	-0.159	-0.160	-0.384	-0.268	-0.328	-0.355	0.088		*0.353*	*0.068*	*-0.035*	*-0.257*	*0.273*
TG (mg/dL)	0.272	0.270	0.270	0.279	-0.025	-0.262	-0.239	0.402	0.269		*-0.069*	*0.140*	*-0.373*	*0.426*
Random Glucose (mg/dL)	**0.596****	**0.596****	**0.596****	0.378	-0.397	**0.676****	-0.047	0.373	-0.200	-0.068		*0.390*	*0.115*	*-0.125*
$AUC_{GLUCOSE}$(mg/dL.min)	**0.462***	**0.462***	**0.462***	0.233	-0.387	0.492	0.030	0.202	-0.134	-0.330	0.358		***-0.719****	*0.548*
K_{ITT} (%/min)	0.198	0.198	0.198	0.270	0.066	0.114	-0.130	0.137	0.208	0.309	0.336	**-0.469***		***-0.851*****
$T½_{GLUCOSE}$ (min)	0.114	0.114	0.114	0.023	-0.148	0.132	0.082	0.250	-0.050	-0.300	-0.022	**0.820****	**-0.682****	

Significant correlations are indicated in **bold** by ** at the 0.01 level and * at the 0.05 level (2-tailed).
Correlations in males are indicated in *italics* .
